# ASCO 2018: highlights in HER2-positive metastatic breast cancer

**DOI:** 10.1007/s12254-018-0441-x

**Published:** 2018-10-11

**Authors:** Rupert Bartsch, Elisabeth Bergen

**Affiliations:** 1 Department of Medicine 1, Clinical Division of Oncology, Medical University of Vienna, Waehringer Guertel 18–20, 1090 Vienna, Austria; 2 Comprehensive Cancer Center Vienna, Medical University of Vienna, Vienna, Austria

**Keywords:** ASCO Annual Meeting 2018, HER2-positive disease, Highlights, Metastatic breast cancer, Review

## Abstract

At the 2018 ASCO Annual Meeting, data from several interesting studies in HER2-positive metastatic breast cancer were presented. While not immediately practice changing, these trials indicate the future directions of drug development in this field. Early phase studies with novel antibody-drug conjugates (ADCs) such as trastuzumab-deruxtecan and trastuzumab-duocarmazine suggest relevant clinical activity of these drugs in pretreated patients; in addition, these ADCs may offer activity in low HER2-expressing tumours as well. ZW25, a bispecific HER2-directed antibody targeting the extracellular domains 2 and 4, showed excellent tolerability and considerable single-agent activity. A combination of T‑DM1 with the tyrosine-kinase inhibitor neratinib yielded high response rates, while a study of trastuzumab plus durvalumab reported disappointing results. Although formally negative, overall survival data from the PHEREXA trial suggest clinical activity of dual HER2-inhibition with trastuzumab and pertuzumab in patients with prior trastuzumab treatment for advanced disease. A combined analysis of two tucatinib studies showed that systemic therapy is active when continued in case of isolated central nervous system progression and stable extracranial disease after local therapy of brain metastases; finally, a small prospective observation in asymptomatic patients with reduced left ventricular ejection fraction suggests that anti-HER2 treatment may be reasonably safe in this population.

## Introduction

Although none of the studies presented at this year’s American Society of Clinical Oncology (ASCO) Annual Meeting in the field of HER2-positive metastatic breast cancer (MBC) are immediately practice changing, several interesting data that add valuable information to the overall picture were presented. In addition, early phase clinical trials with novel agents such as innovative antibody-drug conjugates (ADCs) or bispecific antibodies indicate the future directions of drug development in HER2-positive MBC.

## ADCs and novel antibodies

Currently available HER2-targeted drugs have vastly improved outcomes in patients with HER2-positive MBC; still, resistance will eventually develop in the majority of cases, requiring novel drugs and treatment strategies.

Trastuzumab-deruxtecan (DS-8201a) is an antibody-drug conjugate consisting of trastuzumab and a topoisomerase-I inhibitor [1]. This ADC was designed with a high drug to antibody ratio; in addition, a bystander effect due to its highly membrane-permeable payload was suggested, which may result in antitumour activity against HER2-negative cells. DS-8201a was tested in a phase I dose-escalation trial with multiple expansion cohorts: HER2-positive, trastuzumab-DM1 (T‑DM1) pretreated MBC patients; patients with HER2-positive gastric cancer; MBC patients with low HER2-expression (HER2 1+/2+, *HER2/neu* ISH negative); and a cohort of patients with other HER2-positive or *HER2/neu* mutated solid tumours. In 111 HER2-positive MBC patients, the overall response rate (ORR) was 54.5%. Of note, activity of DS-8201a in the HER2-low breast cancer population (*n* = 34) was comparable (ORR 50%). These results are even more impressive considering the fact that these patients had received a median of 7 and 7.5 prior therapy lines, respectively. Activity was also observed in the other cohorts (ORR gastric cancer 43.2%; ORR other solid tumours 38.7%).

Drug-related adverse events (AE) ≥grade 3 were reported in 41.9% of all patients in the safety population (*n* = 241); the most frequently observed AEs were gastrointestinal (all grade nausea 68.9%, vomiting 34.9%, diarrhoea 26.6%) and haematological (≥grade 3 anaemia 14.9%, neutropenia 15.4%, thrombocytopaenia 10.4%). In addition, five cases of fatal pneumonitis were reported. Therefore, despite relevant clinical activity, further safety evaluation of this drug within the context of larger studies is required.

Another interesting ADC is SYD985, a combination of trastuzumab and the alkylating agent duocarmazine [2]. Again, the drug was tested in a phase I dose-escalation study with expansion cohorts including HER2-positive MBC, HER2-low expressing/hormone-receptor (HR) positive MBC, triple-negative MBC (mTNBC) and other solid cancers with a HER2 expression of a least IHC 1+. Patients were heavily pretreated with a median of six prior treatment lines in all breast cancer subtypes. Of note, 92% of HER2-positive patients had prior exposure to trastuzumab, 80% to T‑DM1, 46% lapatinib and 30% to pertuzumab. ORR in HER2-postive subjects (*n* = 48) was 33% (ORR prior T‑DM1 treatment 29%); progression-free survival (PFS) was 9.4 months (95% CI 4.5–12.5) and 8.3 months (95% CI 4.1–15), respectively. Of note, relevant clinical activity was also observed in HER2-low expressing/HR-positive and mTNBC patients as well (ORR 27% and 40%, respectively), although the PFS in these cohorts was shorter. While treatment was overall well-tolerated, 28 patients (safety population *n* = 146) discontinued treatment due to AEs consisting mainly of eye disorders such as conjunctivitis and keratitis. Grade 1/2 nausea was reported in 20% of patients and grade 1/2 stomatitis in 12%. The only AE ≥grade 3 with an incidence >5% was neutropenia.

Besides ADCs, bispecific antibodies are another area of interest. ZW25 targets two different HER2 domains—ECD4, the trastuzumab-binding domain, and ECD2, the binding domain of pertuzumab [3]. In a dose-escalation study with expansion cohorts, HER2-positive MBC patients with prior exposure to trastuzumab, pertuzumab and T‑DM1 were included. In addition, patients with other HER2-positive solid tumours without remaining standard treatment options and HER2-positive gastroesophageal adenocarcinoma and prior trastuzumab therapy could be included as well. The response rate in the breast cancer cohort (*n* = 20) was 33%, and clinically relevant activity was also observed in HER2-positive gastroesophageal adenocarcinoma and subjects with other HER2-positive solid tumours (ORR 44% and 33%, respectively). Treatment was particularly well tolerated and only one patient experienced grade 3 AEs. There were no dose-limiting toxicities, no treatment-related SAEs or treatment discontinuation due to AEs.

## Novel combination strategies

Based upon the EMILIA trial, the ADC T‑DM1 is considered as the standard-of-care in the second-line treatment of patients with HER2-positive MBC [4]. Activity of the drug, however, seems to be lower after prior exposure to trastuzumab and pertuzumab [5]. Therefore, a phase I trial evaluated the safety and efficacy of combination treatment with T‑DM1 and the second-generation anti-HER2 TKI neratinib [6]. As expected with neratinib, diarrhoea was the dose-limiting toxicity with grade 3 diarrhoea observed in 22%. Activity, on the other hand, was promising, with 12 out of 20 patients experiencing response.

The combination of trastuzumab and immune checkpoint inhibitors (ICPis) is another promising option as this strategy may help to overcome immune-mediated trastuzumab resistance. Fifteen patients with HER2-positive MBC received trastuzumab in combination with the PD-L1 inhibitor durvalumab [7]; again, this was a heavily pretreated population with 15/15 having received prior trastuzumab, 10/15 pertuzumab and 14/15 prior T‑DM1. In addition, 46% of patients had received ≥4 prior lines of chemotherapy. Disappointingly, no response was documented and only four out of 14 evaluable patients had stable disease at 6 weeks. These results are in contrast to data from the KEYNOTE-014 trial, where trastuzumab was combined with pembrolizumab [8]. Of note, in KEYNOTE-014, clinical activity was restricted to the PD-L1 positive population and no response was observed in the PD-L1 negative cohort; in the trastuzumab/durvalumab combination study, 14/15 patients were PD-L1 negative. Therefore, activity of ICPis in HER2-positive MBC may be restricted to patients with PD-L1 positive tumours.

## Response prediction

EGF104900 was a phase III trial randomizing trastuzumab‐pretreated HER2-positive MBC patients to lapatinib or the vertical dual blockade of lapatinib and trastuzumab [9]. In the overall study population, combination treatment improved overall survival (OS) from 9.5 to 14 months (HR 0.74; 95% CI 0.57–0.97; *p* = 0.026). At this year’s ASCO Annual Meeting, a biomarker analysis from this trial was presented [10]. Overall, 177 tumours samples were available; patients with tumours belonging to the HER2-enriched subtype as defined by PAM50 and having high *HER2/neu* mRNA expression (HER2-E/ERBB2H) had a higher response rate, longer PFS (3.5 vs. 1.2 months; HR 0.48; 95% CI 0.34–0.69; *p* < 0.001) and longer OS (14.4 *vs*. 9.1 months; HR 0.65; 95% CI 0.44–0.96; *p* = 0.034). These data are well in line with a combined analysis of neoadjuvant trials of lapatinib plus trastuzumab where the highest pathologic complete response (pCR) rate was observed in the HER2-E/ERBB2H group [11] and Austrian data suggesting that *HER2/neu* gene copy-number correlates with pCR rate [12]. While interesting, these data may not be regarded as practice changing as HER2-positive patients not belonging to the HER2-E/ERBB2H group will likewise receive anti-HER2 therapy as no alternative treatment approach is currently available.

## Clinical practice

PHEREXA was a phase III trial randomizing 452 patients with HER2-positive MBC progressing on or after trastuzumab-based treatment for advanced disease to trastuzumab plus capecitabine or trastuzumab, pertuzumab and capecitabine. As already published, dual HER2 inhibition yielded a nonsignificant prolongation of PFS, which was defined as the primary study endpoint (9 *vs*. 11.1 months; HR 0.82; 95% CI 0.65–1.02; *p* = 0.0731) [13]. The final analysis presented at the 2018 ASCO Annual Meeting reported an OS improvement from 28.1 to 37.2 months (HR 0.76; 95% CI 0.60–0.98) [14]. These data suggest that the benefit of dual HER2-inhibition may not be restricted to the first-line setting but is retained in later treatment lines. Still, PHEREXA is formally a negative study and T‑DM1 therefore remains the standard-of-care in the second-line setting.

Brain metastases (BM) are a common and devastating complication of HER2-positive MBC. Guidelines recommend the continuation of systemic therapy in case of isolated central nervous system (CNS) progression and continued extracranial disease control after local therapy for BM. Of note, only limited clinical data supporting this approach are available. In a combined analysis from two phase Ib trials of tucatinib, a third-generation HER2-TKI, 25 out of 117 patients had an isolated CNS progression [15]; in eleven patients with ongoing tucatinib, the median time to a second progression event was 8.3 months. While patients with extended tucatinib therapy had a more favourable risk profile, these data support the notion that continuing systemic therapy despite newly diagnosed BM provides relevant clinical activity.

Finally, the SAFE-HEaRT study prospectively evaluated the cardiac safety of HER2-directed treatment in 31 asymptomatic patients with left-ventricular ejection fraction (LVEF) ≥40% <50% under optimal cardioprotective treatment including β‑blockers and ACE inhibitors [16]. A cardiac event (defined as symptomatic heart failure or an LVEF drop of ≥10% from baseline and/or ≤35%) was observed in three subjects. This result shows that in selected patients with reduced cardiac function, anti-HER2 treatment is reasonably safe but cardiac events may occur in up to 10% of patients requiring close monitoring.

### Take-home message

- Novel ADCs hold great promise in HER2-positive MBC after progression on current standard options as indicated by high response rates. In addition, drugs such as trastuzumab-deruxtecan and trastuzumab-duocarmazine were also active in HER2-low expressing disease. Regarding tolerability of these drugs, no final conclusion can be drawn and phase II results need to be awaited.
- The bispecific antibody ZW25 was shown to have a favourable toxicity profile and yielded relevant clinical activity.
- Novel combination approaches such as the T‑DM1 plus neratinib may also improve outcome in pretreated patients while activity of immune checkpoint inhibitors may be restricted to PD-L1 positive tumours in patients with HER2-positive MBC.
- Regarding clinical practice, final OS data from the PHEREXA study suggest clinical activity of trastuzumab/pertuzumab combination in trastuzumab-pretreated patients but T‑DM1 remains the second-line standard-of-care.
- A combined analysis of two early phase tucatinib trials support the continuation of systemic therapy after isolated CNS progression and local therapy while the SAFE-HEaRT study indicated that anti-HER2 treatment is reasonably safe in patients with impaired cardiac function.
